# Enhanced Intradermal Delivery of Nanosuspensions of Antifilariasis Drugs Using Dissolving Microneedles: A Proof of Concept Study

**DOI:** 10.3390/pharmaceutics11070346

**Published:** 2019-07-17

**Authors:** Andi Dian Permana, Maelíosa T. C. McCrudden, Ryan F. Donnelly

**Affiliations:** 1 School of Pharmacy, Medical Biology Centre, Queen’s University Belfast, 97 Lisburn Road, Belfast BT9 7BL, UK; 2 Department of Pharmaceutics, Faculty of Pharmacy, Hasanuddin University, Makassar 90234, Indonesia

**Keywords:** doxycycline, albendazole, ivermectin, nanosuspension, microneedles, dermatokinetic, lymphatic filariasis

## Abstract

Conventional oral administration of antifilariasis drugs results in nonspecific targeting of the drugs and the intradermal delivery of nanoparticles with sizes of <100 nm could be used to improve lymphatic uptake. This study investigated the combination of nanosuspension and dissolving microneedles (MN-NS) as an alternative intradermal delivery approach for the delivery of antifilariasis drugs, namely doxycycline, albendazole, and ivermectin. NS were fabricated and optimized using a bottom-up technique. The NS were then incorporated into the MN arrays. The optimized NS were <100 nm in diameter. Furthermore, MN-NS had suitable mechanical strength and insertion capabilities. The dermatokinetic study revealed that the delivery of drugs into the dermis of excised neonatal porcine skin by MNs was significantly higher than that from a needle-free patch, with 29.29 ± 4.65%, 31.54 ± 5.35%, and 34.54 ± 4.98% of doxycycline, albendazole sulfoxide, and ivermectin retained in the dermis after 24 h. The results presented here serve as proof of concept for the significant enhancement of drug retention times in the dermis, following their formulation into NS and delivery *via* MN. Leading on from these studies, future work must investigate in vivo lymphatic pharmacokinetic profiling of drugs formulated into NS, in a suitable animal model.

## 1. Introduction

Lymphatic filariasis (LF) is a human parasitic disease caused by the *Filarioidea* family of nematodes, triggering damage to the lymphatic system and leading to elephantiasis, hydrocele, and kidney damage (including chyluria) [1,2]. Nematodes are transmitted to individuals who are bitten by infected mosquitoes. *Aedes*, *Anopheles*, *Culex*, or *Mansonia* are the species of mosquitoes that act as intermediate vectors and hosts of the filarial nematodes. Adult nematodes reside in the lymphatic vessels and lymph nodes of infected individuals where they can dwell for several years [3]. The endosymbiotic bacteria, *Wolbachia*, has been found to live in filarial nematodes. These bacteria have a major role in the biological life cycles of the nematodes by helping the reproduction and survival of the filarial nematodes [4]. In 2017, it was reported that 51 countries were still considered to require mass drug administration (MDA) for LF treatment [1]. The World Health Organization (WHO) has established LF as one of the world’s leading causes of long-term and permanent disability [1,5].

The current MDA approach to combat LF involves the delivery of a combination of several antifilariasis drugs/medications. In this approach, albendazole (ABZ), with ivermectin (IVM) are routinely administered orally to target and kill the nematodes [5,6]. Moreover, the use of doxycycline (DOX) to target *Wolbachia* has shown a significant decrease in the number of *Wolbachia* and microfilariae in the bloodstream in a human trial [7]. However, despite the fact that these drugs effectively reduce the viability of microfilariae in the bloodstream, they have limited effects on adult filarial in the lymph nodes [8]. When administered *via* this route, the drugs must pass across the epithelium of the intestine in order to reach the underlying interstitial space drained by the lymph and blood capillaries [9]. As a result, drug concentrations in the lymphatics, the anatomical site where the adult nematodes dwell and where drug should be targeted, are severely limited. Consequently, the development of a novel antifilarial drug formulation and delivery system that could be utilized to target delivery of the drugs to the site of action, would increase the concentrations of drugs at these sites and thus the effectiveness of the treatment [10].

Several emerging technologies have been applied in lymph targeting, namely liposomes [11], polymeric nanoparticles [12], solid lipid nanoparticles [13], self-emulsifying self-nanosuspension drug delivery systems [14], nanosuspensions (NS) [15], and microneedle (MN) array delivery systems [16]. NS are unique liquid sub-micron colloidal dispersions of pure drug particles stabilized using an appropriate surfactant and/or polymer and typically have particle sizes of 1–1000 nm [17]. NS technologies are being actively investigated in lymph targeting of a variety of anti-HIV drugs: lopinavir (LPV), ritonavir, tenofovir (TFV), and lamivudine (3TC) [15]. DOX, ABZ, and IVM are good candidates of this approach due to their poor solubility. To elaborate on one such novel approach, the use of NS is undergoing considerable investigation in the production of small particle sizes of poorly soluble drugs [18]. With respect to the potential route of administration of drugs to specifically target the lymphatic system, the intradermal route has been found to be the most appropriate route to deliver drug into the lymph due to higher lymph flow rates in the skin compared to other interstitial sites [9]. It has been reported that due to the characteristics of lymph capillaries, after the administration at this site, particles with sizes between 10 and 100 nm are taken up by the lymphatic capillaries, while particles of less than 10 nm are absorbed by blood capillaries [19]. In contrast, particles of more than 100 nm are retained at the administration site [19]. However, the administration of drugs *via* hypodermic needles intradermally could result in several disadvantages, namely pain, needle phobia, poor patient compliance, and the generation of hazardous biological waste [20]. In addition to this, in spite of its advantages, intradermal injections are not generally used clinically and it has been reported that administration of intradermal injection by trained professionals are not always successful [21]. MNs are minimally invasive devices which can by-pass the *stratum corneum*, the principal skin barrier to topically-applied drugs, without pain and, as such, are envisioned as novel trans- and intra-dermal drug delivery systems [22]. MNs are applied to the skin surface and form small aqueous pores through which drugs penetrate to the dermal microcirculation [22,23]. With a view to exploiting this novel delivery system in the administration of NS, a number of studies have already evaluated the lymphatic uptake of drugs using MNs as intradermal delivery vehicles. These studies have shown increases in drug concentration in the lymph nodes compared to the intravenous and subcutaneous delivery routes [16,24]. Furthermore, our research group has previously developed MN formulations in combination with NS to deliver cholecalciferol [25] and rilpivirine [26] intradermally. With reference to the latter study, using this combinatorial delivery approach, rilpivirine was detected in the lymph nodes of treated rats, providing justification for the continued exploration of this approach [26]. Leading on from this, the use of drug NS with particle sizes of ~100 nm, in combination with intradermal delivery, could potentially be used to target nematode infection sites in lymph nodes for the treatment of LF.

In the present study, we outline the combinatorial approach of using drugs in NS form, with intradermal delivery of the NS *via* MN, using an antibacterial drug (DOX) and two antifilariasis drugs (ABZ and IVM). As the intradermal route prevents drugs undergoing first-pass metabolism in the liver, albendazole sulfoxide (ABZ-OX), as an active metabolite of ABZ, was used in this study. We developed and optimized the drug NS using central composite design (CCD) and incorporated the optimized NS into dissolving MNs. Following mechanical characterization studies of the MN, novel ex vivo dermatokinetic studies, skin distribution, and deposition studies were carried out. The outcomes of these proof of principle studies allowed us to form insights into the kinetic profiles of the drugs in the epidermis and dermis, over the course of different application durations. The results presented here point towards the potential to retain NS in the dermis layer which could potentially have positive implications for lymphatic uptake of appropriately formulated drugs.

## 2. Materials and Methods

### 2.1. Chemicals and Materials

Doxycycline monohydrate (DOX) (purity, ≥98%) and ivermectin (purity, ≥98%) of analytical grade were purchased from Alfa Aesar (Lancashire, UK), while chloroform, citric acid, dimethylsulfoxide (DMSO), albendazole sulfoxide (ABZ-OX) (purity, ≥99.9%), hydroxyl propyl methylcellulose (HPMC), poly (vinyl alcohol) (PVA) (MW 9–10 kDa), poly (vinyl alcohol) (PVA) (31–50 kDa), sodium carboxymethylcellulose (NaCMC), sodium hydroxide, and sodium lauryl sulphate (SLS) were purchased from Sigma-Aldrich (Dorset, UK). Poly (vinyl pyrrolidone) (PVP) (58 kDa) was obtained from Ashland (Kidderminster, UK). Pluronic^®^ F127 (PF127), Pluronic^®^ F108 (PF108), Pluronic^®^ F68 (PF68) were obtained from BASF SE (Ludwigshafen, Germany). Ultrapure water was obtained from a water purification system (Elga PURELAB DV 25, Veolia Water Systems, Dublin Ireland). All other reagents were of analytical grade and purchased from standard commercial suppliers.

### 2.2. Preparation of NS

The NS were prepared using a bottom-up technique. Two methods were applied, namely the solvent-antisolvent [27] and acid-base neutralization precipitation method [28] under sonication (Davidson and Hardy Ltd. Cooperating with Fisher Scientific, Leicestershire, UK), with some modifications. The solvent-antisolvent precipitation method was employed to formulate DOX and IVM NS. ABZ-OX NS were prepared using acid-base neutralisation precipitation. Several polymers and surfactants, including PVP, PVA (MW 9–10 kDa), NaCMC, HPMC, PF127, PF108, PF68, and SLS were investigated as stabilizers.

#### 2.2.1. Doxycycline Nanosuspensions (DOX NS)

In DOX NS preparation, DMSO was used as a solvent and water containing different concentrations of stabilizers was used as an antisolvent phase. Initially, doxycycline was dissolved in 1 mL of DMSO and following this, the drug solution was dropped into 5 mL of antisolvent phase in the probe sonicator (an amplitude of 80% with 10 s pulse on and 5 s pulse off). All the processes were performed in an ice bath.

#### 2.2.2. Albendazole Sulfoxide Nanosuspensions (ABZ-OX NS)

With respect to ABZ-OX NS preparation, the drug was dissolved in 1 mL of 0.5 M sodium hydroxide solution. The drug solution was precipitated in the probe sonicator (an amplitude of 80% with 10 s pulse on and 5 s pulse off) by adding the dissolved drug into 5 mL of 0.3 M citric acid solution containing stabilizers. The NS production was carried out in an ice bath.

#### 2.2.3. Ivermectin Nanosuspensions (IVM NS)

In order to formulate IVM NS, the solvent phase was prepared by dissolving IVM in 0.5 mL of chloroform, while the antisolvent phase was prepared by dissolving the stabilizers in water. Following this process, the solvent phase was added into 5 mL of the antisolvent in the probe sonicator (an amplitude of 80% with 10 s pulse on and 5 s pulse off) on an ice bath. Chloroform in the formulation was removed by stirring the formulation on a magnetic stirrer for 2 h.

**Figure 1:**
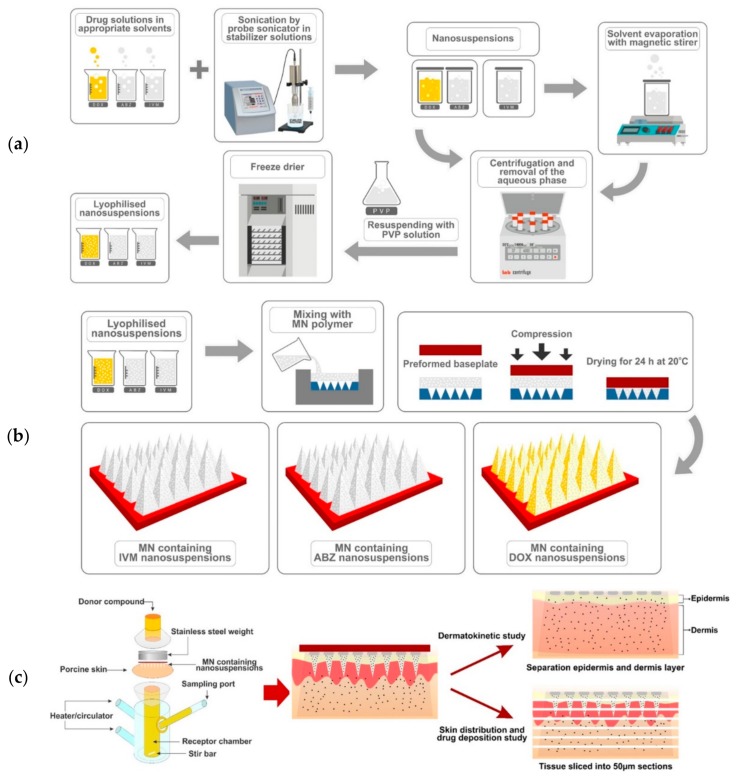
(**a**) Schematic representation of nanosuspension (NS) preparation; (**b**) microneedle (MN) array preparation; and (**c**) dermatokinetic, skin distribution and deposition studies.

In an attempt to remove the residual DMSO from the DOX NS and the stabilizers from all other NS, the NS were centrifuged (Sigma^®^ 1–14 micro-centrifuge, SciQuip Ltd., Shropshire, UK) at 14,000× *g* for 30 min and washed three times with purified water. Prior to the lyophilization process, 5 mL of cryoprotectant solution (2.5% *w*/*v* of PVP) was added into the free-surfactant NS, as described previously. These mixtures were pre-frozen at −80 °C in an ultra-low temperature freezer for 2 h, then transferred to the freeze dryer (Virtis Advantage Bench-top Freeze-drier system, SP Scientific, Warminster, PA, USA) for 26 h to produce dry powder particles. The preparation of NS is described in Figure 1a.

### 2.3. Optimization of NS Formulations

Optimization of NS formulations was performed using a CCD with Design Expert Software version 11 (State-Ease, Minneapolis, MN, USA). Four main factors which affect NS characteristics were chosen, namely drug concentration, stabilizer concentration, antisolvent volume, and sonication time. These factors were analyzed on the software at five different levels (−alpha, low, medium, high, +alpha) and the three responses were then recorded, namely particle size, PDI, and zeta potential. The level of drug concentration, antisolvent volume and sonication time were similar in all optimizations. While stabilizer concentration of DOX formulation was different from the ABZ-OX and IVM formulations. The optimization of the experimental design for the three drugs are shown in Table S1.

### 2.4. Characterization of Optimized NS

The determinations of particle sizes, polydispersity indexes (PDI), and zeta potentials of the various NS were performed using NanoBrook Omni particle sizer and zeta potential analyzer (Brookhaven, New York, NY, USA). The particle size was expressed in intensity. The morphologies of the NS were examined using the transmission electron microscope (TEM) (JEM-1400Plus; JEOL, Tokyo, Japan). Moreover, Fourier transform infrared (FTIR) studies for pure drugs and NS was carried out using FTIR spectrometer (Accutrac FT/IR-4100™ Series, Jasco, Essex, UK). Differential scanning calorimetry (DSC) for the pure drugs, physical mixture, the NS was performed using a differential scanning calorimeter (DSC 2920, TA Instruments, Surry, UK). Finally, the drug contents of each formulation were analyzed using the validated high-performance liquid chromatography (HPLC) method described in the analytical section.

### 2.5. In Vitro Drug Release Study

In vitro release of DOX, ABZ-OX and IVM from NS were carried out using dialysis method [29]. In order to achieve sink conditions, different release media were used based on the solubility of the drugs. In this study, PBS (pH 7.4), PBS:ethanol (70:30, *v*/*v*) and PBS:methanol (50:50, *v*/*v*) were used as the release media for DOX, ABZ-OX, and IVM, respectively. The study was conducted using the pure drug and the lyophilized NS (equivalent to 5 mg of each drug). The NS (1 mL) was dispersed into the Spectra-Por^®^, 12,000–14,000 MWCO dialysis membrane (Spectrum Medical Industries, Los Angeles, CA, USA). The bags were each suspended in 100 mL of release media at 37 °C in an orbital shaker at 100 rpm. At predetermined time intervals, aliquots of 1 mL of sample were taken and replaced by 1 mL fresh release media. To calculate the amount of drug released, the samples were then analyzed using the HPLC method, as described in the analytical section.

The cumulative drug release was further fitted to different mathematical kinetic models, as outlined in previous studies: [30]

$$\mathrm{Zero}\text{-}\mathrm{order}:\;Q_{t}=Q_{0}+K_{0}t$$

$$\mathrm{Zero}\text{-}\mathrm{order}:\;Q_{t}=Q_{0}+K_{0}t$$

$$\mathrm{First}\text{-}\mathrm{order}:\;lnQ_{t}=lnQ_{0}+K_{1}t$$

$$\mathrm{Higuchi}:\;Q_{t}=\;K_{H}\sqrt{t}$$

$$\mathrm{Korsmeyer}\text{-}\mathrm{Peppas}:\;Q_{t}=K_{t}n$$

$$\mathrm{Hixson}\text{-}\mathrm{Crowell}:\;Q_{0}^{1/3}-Q_{t}^{1/3}=\;K_{s}t$$

where $Q_{t}$ (%) is the percentage of drug released at the time t, $Q_{0}$ is the starting value of $Q_{t},$ t is the time, n is the diffusion release exponent, $K_{0}$, $K_{1}$, $K_{H}$, $K_{t},$ and $K_{s}$ are the release constants corresponding to relevant kinetic models. The model parameters were calculated using DDsolver [31].

### 2.6. Two-Step Casting of MN and Needle-Free Patch

Several formulations were prepared separately in order to optimize the MNs for delivery of the DOX, ABZ-OX, and IVM in NS. Various aqueous gel formulations were prepared using selected polymers of various concentrations. The preparation of MNs is described in Figure 1b. Six different formulations were prepared, consisting of 20% *w*/*w* of lyophilized NS of drugs and 50% *w*/*w* of PVP (formulation code A in each formulation); 30% of lyophilized NS of drugs and 50% of PVP (formulation code B in each formulation); 20% *w*/*w* of lyophilized NS of drugs and 15% *w*/*w* of PVA (formulation code C in each formulation); 30% *w*/*w* of lyophilized NS of drugs and 15% *w*/*w* of PVA (formulation code D in each formulation); 30% *w*/*w* of lyophilized NS of drugs and 15% *w*/*w* of PVA and 5% *w*/*w* of PVP (formulation code E in DOX formulation); 40% *w*/*w* of lyophilized NS of drugs and 15% *w*/*w* of PVA and 10% *w*/*w* of PVP (formulation code F in each formulation). Initially, the lyophilized NS were added to the aqueous formulations of selected polymers and mixed until homogenous. Following this, 100 mg of the aqueous blend was then poured into the MN molds, manufactured using injection molding, (needle density of 19 × 19, pyramidal needles; 500 μm height and 300 μm width at base and 300 μm interspacing). A pre-cast dry baseplate (1 × 1 cm^2^), prepared from 15% *w*/*w* PVP (MW 360 kDa) and 1.5% *w*/*w* glycerol was placed behind the needles. Finally, the formulations were placed in a positive pressure chamber and a pressure of 3–4 bar was applied for 15 min to fill the cavity of the molds. Lastly, the MNs were dried at room temperature for 24 h and were removed from the molds. The MNs were visually examined using a Leica EZ4D digital light microscope (Leica Microsystems, Milton Keynes, UK) and scanning electron microscope (SEM) TM3030 (Hitachi, Krefeld, Germany). For the needle-free patch preparation, similar formulations were prepared. The aqueous blend (100 mg) was placed on the top of a flat silicon sheet. Afterwards, a pre-cast dry baseplate was attached behind the formulations and these were allowed to dry at room temperature for 24 h.

### 2.7. Skin Sample Collection

Excised skin from stillborn piglets was used in ex vivo studies, with full-thickness skin samples having been obtained less than 24 h post-mortem. Skin samples were kept in sealed petri dishes at −20 °C until use. Prior to the commencement of experiments, hair was removed from the skin using a disposable razor and the skin was then equilibrated in phosphate buffered saline (PBS), pH 7.4, for 30 min.

### 2.8. Mechanical and Insertion Properties of MNs

Mechanical testing was performed using a TA-TX2 Texture Analyzer (Stable Microsystem, Haslmere, UK), as described previously [32]. In order to evaluate the insertion ability of the MNs, Parafilm^®^M was used as an artificial skin layer for the MNs insertion study as reported in a previous study [33]. The insertion ability of the MNs in Parafilm^®^M and full-thickness porcine skin were also evaluated using an optical coherence tomography (OCT) microscope (Michelson Diagnostics Ltd., Kent, UK), as outlined in previous study [32]. The height of needles inserted was measured using the imaging software, ImageJ^®^ (National Institute of Health, Bethesda, MD, USA).

### 2.9. Calculation of Theoretical Drug Content Localized to the Needles

Initially, baseplates were prepared using the optimized formulation of the MN to calculate the density of the formulation. The drug amount (mg) in the needle tips were calculated using the following equation [26]:

$$\mathrm{Drug}\;\mathrm{in}\;\mathrm{the}\;\mathrm{MN}\;\mathrm{tips}:\;N\;\times \;\frac{\left(h.a^{2}\right).\rho .\left[drug\right]}{3}$$

where *N* is the number of needle tips (361 tips), *a*^2^ is the width of base of the tips (0.3 mm), h is the height of the tips (0.5 mm), ρ is the density of the dry formulation, and [drug] is the concentration of drugs in dry formulation (mg drug/mg NS).

### 2.10. In Vitro Dermatokinetic, Skin Distribution and Deposition Studies

These studies were carried out using Franz diffusion cells [34]. Full-thickness skin used in these studies were affixed to the Franz diffusion cells using cyanoacrylate glue. PBS pH 7.4 was used in the receiver compartment and was thermostatically held at 37 ± 1 °C with stirring at 600 rpm. The MNs arrays were then inserted into the skin using manual force for 30 s and a circular stainless-steel weight of 5.0 g placed on top to hold the MNs in place. The donor compartment and sampling arm was wrapped using Parafilm M^®^. The illustration of this study is shown in Figure 1c.

In the dermatokinetic studies, at the requisite sampling time points (15 min, 30 min, 45 min, 1.5 h, 1 h, 2 h, 3 h, 4 h, 5 h, 6 h, and 24 h), the MNs were removed from the full-thickness skin and the skin was washed three times with deionized water to remove any excess formulation. Skin sections were then collected using a biopsy punch (5 mm diameter) (Stiefel, Middlesex, UK). The skin was then placed into a 1.5 mL microtube and samples were heated in a water bath at 60 °C for 2–3 min. The dermal layers were further manually separated from the epidermis layers using tweezers. Then, 1 mL methanol was added into each layer and each mixture was homogenized for 10 min at 50 Hz using Tissue Lyser LT (Qiagen, Ltd., Manchester, UK) to extract the drug from the skin. Finally, a sample of supernatant was then collected and analyzed using the HPLC method, as described in Section 2.11. The data obtained were then fitted to a one-compartment open model using PK Solver software [35]. The curve of drug concentration versus time was made and the mean peak drug concentration (*C*_max_), the time of *C*_max_ (*t*_max_), and the area under curve from time 0 h to 24 h (AUC_0-t_) were all calculated.

In skin distribution and deposition studies, the skin samples were collected at respective sampling time points. The time points of 1 h, dermis *t*_max_ from dermatokinetic study and 24 h were selected for this study. The samples were collected using a similar procedure to that described previously. Following this, the samples were flash frozen in liquid nitrogen. The biopsied samples were then sectioned into 50 μm sections using a Leica CM1900 Cryostat (Leica Microsystems, Nussloch, Germany) and four consecutive slices were collected into microtubes. This process was continued until the whole skin was sliced. In order to analyze the amount of drug distributed and deposited into each slice, 300 μL of methanol was added to the tissue sections. Each sample was vortexed for 30 min to dissolve the drug and the sample was then centrifuged at 14,000× *g*, 15 min. A sample of supernatant was then collected and analyzed using the HPLC method, as described in in the analytical section.

### 2.11. Instrumentation and Chromatographic Condition for Analytical Method

The quantifications of DOX, ABZ-OX, and IVM were performed separately using reversed-phase HPLC with Phenomenex^®^ Luna C_18_ (ODS1) column (150 mm × 4.6 mm i.d. with 5 μm particle size) using an Agilent Technologies 1220 Infinity compacted LC Series consisting of Agilent degasser, Binary Pump, auto standard injector and Detector (Agilent Technologies UK Ltd., Stockport, UK). The mobile phases used in all analysis processes were 25 mM sodium dihydrogen phosphate buffer (with 0.1% *v*/*v* TEA, pH 2.5 adjusted using orthophosphoric acid) and methanol (70:30 *v*/*v*) for DOX; 25 mM sodium dihydrogen phosphate buffer (with 0.1% *v*/*v* TEA, pH 3 adjusted using orthophosphoric acid), and methanol (75:25 *v*/*v*) for ABZ-OX; and 0.1% *v*/*v* of trifluoroacetic acid in water and methanol (5:95 *v*/*v*) for IVM. The injection volume and flow rate were 25 μL and 1 mL/min, respectively and the analyses were performed at room temperature with UV detection of 271 nm, 295 nm, and 245 nm for DOX, ABZ-OX, and IVM, respectively. The chromatograms were analyzed using Agilent ChemStation^®^ Software B.02.01. The analytical methods were validated as per the International Committee of Harmonization (ICH) 2005.

### 2.12. Statistical Analysis

The results were expressed as means ± standard deviation (SD) of the mean. The data were analysed using GraphPad Prism^®^ version 6 (GraphPad Software, San Diego, CA, USA). The Mann-Whitney U test was performed for comparison between drug NS and pure drug, in terms of the drug release after 24 h, and between MN and needle-free patches, in terms of dermatokinetic parameters of drugs. The Kruskal-Wallis test with post-hoc Dunn’s test was used for comparison of the mechanical strength of MNs of all formulations. In all cases, *p*-value < 0.05 was considered as a significant difference.

## 3. Results and Discussion

### 3.1. Preparation of NS and Screening of Stabilizers

In DOX NS preparation, different polymers and surfactants, at a variety of different concentrations were utilized to produce the NS. Our preliminary studies (Figures S1–S3) exhibited that the NS prepared from PVP and SLS exhibited smaller particle size compared to the NS prepared from other stabilizers. Therefore, the combination of PVP and SLS as stabilizers was selected for the optimization process. The optimization was performed using central composite design (CCD). In the optimization process, SLS concentration was kept constant at 0.2% *w*/*v*. Whereas, drug concentration, PVP concentration, antisolvent volume and sonication time were optimized. The software suggested 24 formulations for the optimization and the responses of dependent variables in NS formulations are exhibited in Table S2. The results showed that the particle size, PDI, and zeta potential of DOX NS were in the range of 85.4–1136.3 nm, 0.20–0.83, and –23.43 to –31.32 mV, respectively. Depending upon electrostatic attraction, the combination of PVP and SLS leads to the formation of a shortened polymer-surfactant double protecting layer, leading to smaller particle generation [36]. Based on the fit statistical analysis results, the difference of all predicted R^2^ from adjusted R^2^ was less than 0.2. Although the results exhibit large variability in the particle size results, the *p*-value of the model was <0.0001. In addition, there were no other recommendations for the transformation of the data prior to the analysis. The adeq precision was 17.0647, 13.9614, and 33.5281 for particle size, PDI, and zeta potential, respectively. As per the requirement in the software, values greater than four are desirable. Accordingly, this model was used in order to design the optimum formula for DOX NX. The results of fit statistical analysis for all responses of DOX NS formulation are shown in Table S5.

With respect to ABZ-OX and IVM NS formulations, PF127 solution was found to be an appropriate stabilizer. The CCD was again used in optimization process. The optimization was carried out for drug concentration, PF127 concentration, antisolvent volume, and sonication time. In this optimization, 24 formulations were also suggested by the software and the responses of dependent variables in ABZ-OX and IVM NS formulations are exhibited in Tables S3 and S4, respectively. The results showed that the particle size of ABZ-OX and IVM NS were in the range of 84.2–1112.4 nm and 87.8–1168.0 nm, respectively. PDI values obtained were in the range of 0.19–0.76 and 0.20–0.83 for ABZ-OX and IVM NS, respectively. The zeta potentials were ranging from −12.36 to −19.05 mV, and −12.98 to −20.25 mV in the case of ABZ-OX and IVM NS, respectively. The stabilization of NS by PF127 may be attributed to its poly (propylene oxide) (PPO) and poly (ethylene oxide) (PEO) chains [37]. The hydrophobic PPO chains of Pluronic promotes the polymer to adsorb onto the surface of hydrophobic drugs [38], while the hydrophilic PEO chains extend into the aqueous phase, providing steric stabilization by inhibiting the aggregation [39]. Based on the fit statistical analysis results, the difference of all predicted *R*^2^ from adjusted R^2^ was less than 0.2. As explained in the DOX NS optimization, the *p*-value of the model was also found to be <0.0001, despite the variability of the particle size results and no recommendation of the data transformation was required before the analysis of the data. In addition, the adeq precision of all designs was greater than four, which were in the range of 13.9614–33.5281. Therefore, these models were used in order to design the optimum formulae. The results of fit statistical analysis for ABZ-OX and IVM NS formulations are shown in Table S5.

### 3.2. Optimization of NS Formulations

#### 3.2.1. Effect of the Parameters on the Particle Size and PDI

**Figure 2:**
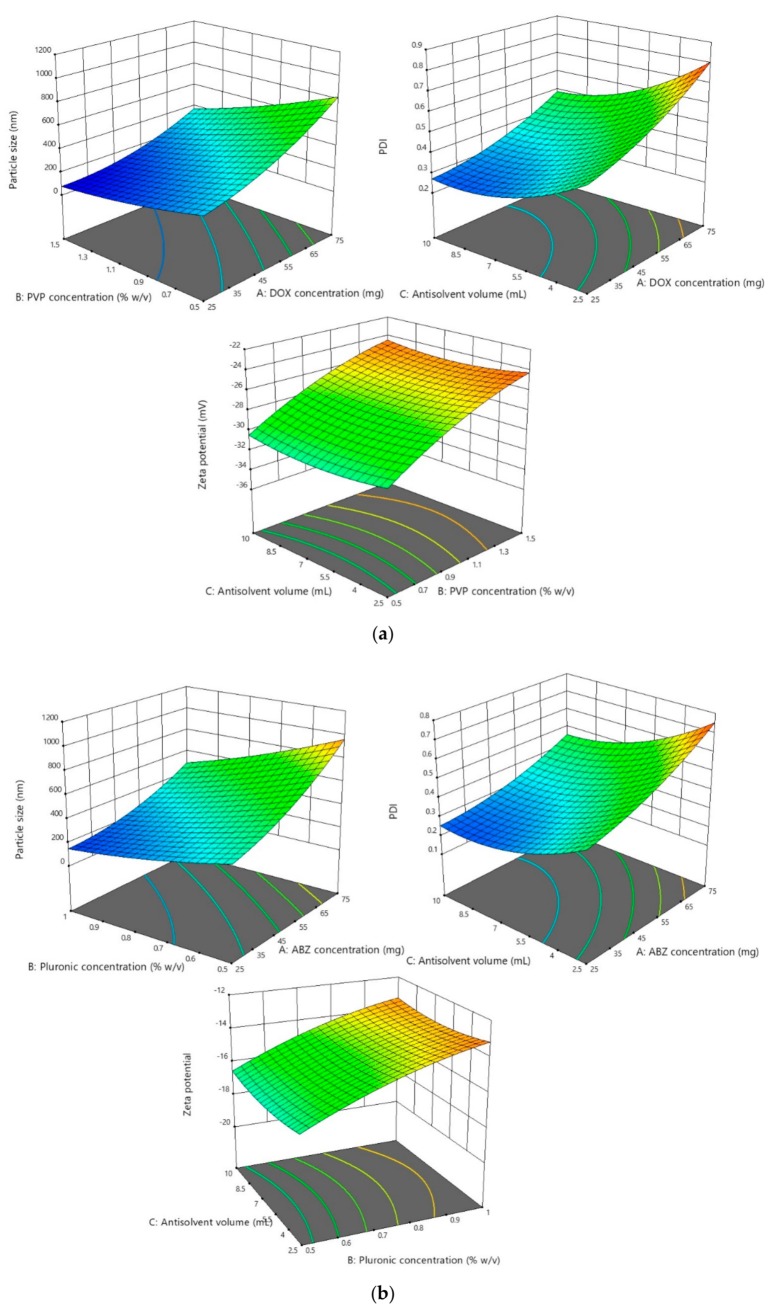

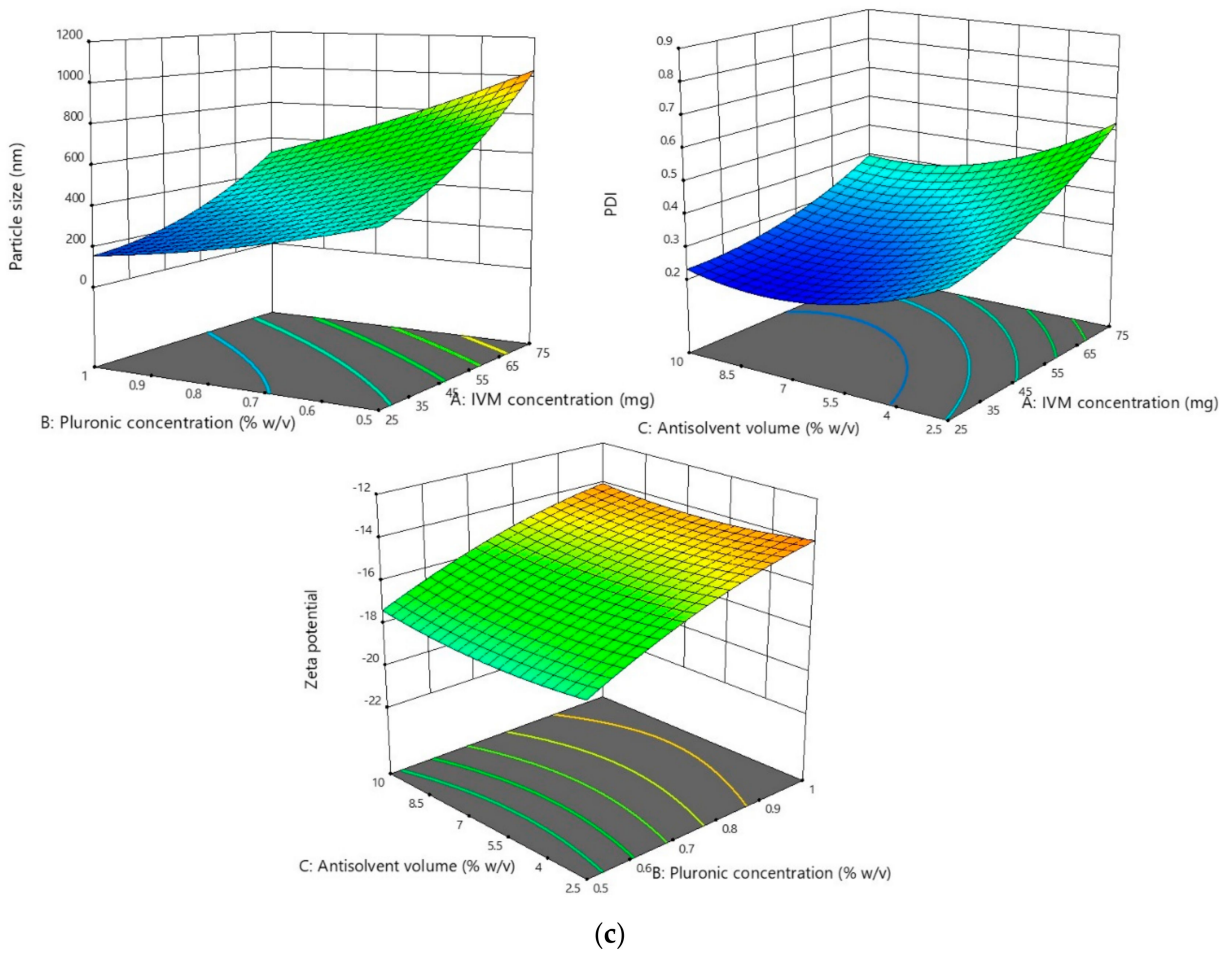
(**a**) Representative response surface plots describing the effect of dependent variables on the particle size, polydispersity indexes (PDI), and zeta potential of doxycycline (DOX) NS. (**b**) Representative response surface plots describing the effect of dependent variables on the particle size, PDI, and zeta potential of albendazole sulfoxide (ABZ-OX) NS. (**c**) Representative response surface plots describing the effect of dependent variables on the particle size, PDI, and zeta potential of ivermectin (IVM) NS.

The results showed that the particle size and PDI of all optimization processes followed the quadratic model. In terms of the particle size analysis, the F-value was found to be 20.77, 19.85, and 19.55 for DOX, ABZ-OX, and IVM NS, respectively. The F-value of 14.83, 15.72, and 15.68 were obtained in PDI analysis for DOX, ABZ-OX, and IVM NS, respectively. Moreover, the *p*-value of <0.001 was found in all cases, indicating that the factors selected had a significant effect on the particle size and PDI. The representative 3D graphs describing the effect of selected parameters on the particle sizes and PDI are shown in Figure 2.

In terms of parameters observed, drug concentrations were found to have a significant effect on the particle size and PDI (*p*-value < 0.001). The results exhibited that the increase in drug concentration was followed by the increase of particle size and PDI. This might be caused by the drugs supersaturation phenomenon. High supersaturation was needed in order to produce small and uniform particles to accelerate a nucleation rate greater than the rate of crystal growth. However, when the drug concentration was excessive, a large number of nuclei quickly formed on the interface of solid phase and liquid phase and slowed the diffusion from solvent to antisolvent, leading to particle adhesion and larger particle production [40]. Furthermore, the increasing drug concentration may cause the increasing of the viscosity of the solutions and avoiding particle diffusion [41]. The concentration of PVP and PF127 as stabilizers also had a significant effect (*p*-value < 0.001) on particle size and PDI. The results exhibited that low concentration of stabilizers produced larger particles, potentially caused by an inadequate amount of stabilizer coating the hydrophobic surfaces of the drugs [27].

The effect of antisolvent volume was also studied. As shown in Tables S2–S4, the increase in antisolvent volume was followed by decrease of particle size and PDI. Throughout precipitation, the growth occurs at the same time as the formation of nuclei. Therefore, the increase of the volume of antisolvent decreased the concentration of particle in the liquid phase and hence prevented the possibility of agglomeration between each particle in the suspension, contributing to small particle formation [42]. In addition, it was found that in order to form a small particle, the ABZ-OX and IVM NS required less antisolvent volume compared to DOX NS. This may be due to the difference in viscosity of the solvents used to dissolve the drugs. DMSO has a higher viscosity than chloroform or 0.5 M sodium hydroxide solution. The higher viscosity may avoid particle diffusion to form the small particles [41]. Accordingly, a greater antisolvent volume was required to form smaller particles.

In NS formulation, duration of sonication also plays a major role in the forming of smaller particle sizes. In this study, it was found that the particle sizes of the NS decreased following an increase in sonication time as a result of the greater use of energy [43]. However, despite the decrease of particle size, the increase in sonication time did not exhibit significant effect (*p*-value > 0.05) on the particle size of NS.

#### 3.2.2. Effect of the Parameters on Zeta Potential

The results showed that the zeta potential of all optimization processes followed the quadratic model. With respect to the analysis results, the F-values of 100.30, 96.56, and 68.34 were obtained for DOX, ABZ-OX, and IVM, respectively. Additionally, the *p*-values were found to be <0.001 in all cases, demonstrating that the model was significant. The effects of selected parameters on the zeta potentials of the NS are illustrated in the 3D graphs in Figure 2. The only factor to shown a significant effect on the zeta potential of the NS was the stabilizer concentration. In DOX formulation, which contained SLS 0.2% *w*/*v*, the increasing concentration of PVP decreased the value of zeta potential. It is well-known that SLS is an anionic surfactant with negative charge [44]. Therefore, the increase in PVP could possibly decrease the charge of dispersions containing SLS. In the ABZ-OX and IVM formulations, the same trend was observed. PF127 has a higher number of PPO units, resulting in higher thickness of adsorbed polymer, thus lowering the value of the zeta potential. The decrease of zeta potential might be caused by the outer shift of the slipping plane, which is extremely crucial in zeta potential measurement [39].

### 3.3. Characterization of Optimized NS

Based on the analysis of the software outputs, optimized formulations were selected for further investigation. The criteria were set in order to achieve a particle size of less than 100 nm with high drug content in the formulations. Several solutions were given by the software and ranked based on the desirability factor. One optimum formulation for each of DOX, ABZ-OX, and IVM was selected and the NS from the optimization recommendation were prepared in three batches. The high desirability of the optimum formula was found in these optimizations, which were 0.99, 0.98, and 0.99 for DOX, ABZ-OX, and IVM NS, respectively. The particle sizes of the optimized formulation were 98.87 ± 9.77 nm, 96.53 ± 8.43 nm, and 98.12 ± 7.76 nm for DOX, ABZ-OX, and IVM, respectively. The comparison between the predicted values and the actual values is shown in Table S6. The results revealed that the bias between predicted values and the actual values was ±15%, indicating that the computationally designed optimization processes had been successful. Regarding the drug content, it was found that the drug contents of the optimized NS were 0.243 ± 0.03 mg/mg, 0.252 ± 0.02 mg/mg, and 0.249 ± 0.03 mg/mg of lyophilized NS for DOX, ABZ-OX, and IVM formulations, respectively.

**Figure 3:**
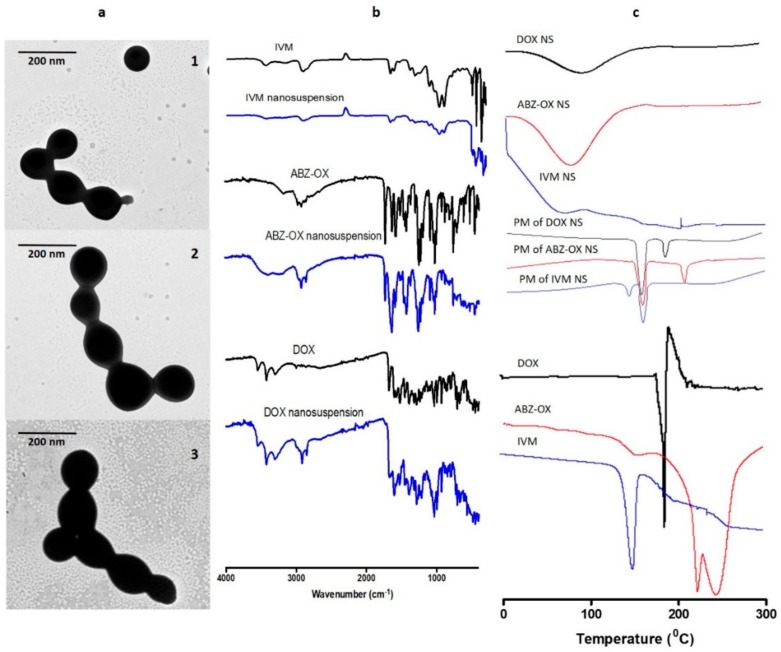
Transmission electron microscope (TEM) images of the NS of (**a.1**) DOX; (**a.2**) ABZ-OX; and (**a.3**) IVM at a magnification power of 30,000×. (**b**) Nanoparticles were imaged using a JEOL JEM-1400Plus transmission electron microscope, kV = 80. Fourier transform infrared (FTIR) spectra of DOX, ABZ-OX, IVM, and their NS formulations. (**c**) Differential scanning calorimetry (DSC) thermogram of DOX, ABZ-OX, IVM, and their respective physical mixture (PM) and NS formulations.

The morphologies of the optimized NS observed by TEM are shown in Figure 3a. These micrographs showed that the DOX, ABZ-OX, and IVM NS produced were all spherical in shape. The size of these NS obtained from TEM were ~100 nm, which was in close agreement with the size obtained by the particle size analyzer.

FTIR analysis (Figure 3b) revealed that DOX showed a major peak at 1527 cm^−1^ due to aromatic N–H bending. Multiple characteristic peaks were also observed between 1397 cm^−1^ and 1682 cm^−1^ due to aromatic C=O and C=C stretching. Sharp peaks were observed at 2917 cm^−1^, 3308 cm^−1^, and 3428 cm^−1^, corresponding to C–H stretching, primary N–H group and primary O–H groups, respectively. The characteristic peak of ABZ-OX was observed at 1027 cm^−1^ due to S=O bonding. Moreover, peaks at 1519 cm^−1^, 1729 cm^−1^, and 2972 cm^−1^ were corresponding to C=N stretching, –COO bending and C–H stretching, respectively. In IVM spectra, the specific peaks were found at 1186 cm^−1^, 1715 cm^−1^, 1729 cm^−1^, 2949 cm^−1^, and 3476 cm^−1^, relating to C–O–C group, C=O bending, aliphatic ketone C=O, axial deformation of free C-H and free O–H, respectively. The characteristic bands of DOX, ABZ-OX, and IVM were also observed in FTIR spectra of NS formulations. The presence of all major functional groups of all drugs in the final formulation confirmed that there were no chemical interactions between pure drugs and any of the excipients used.

Figure 3c depicts the thermogram profiles of DOX, ABZ-OX, IVM, and their respective NS formulations. The sharp endothermic peaks at 168 °C in DOX, 226 °C in ABZ-OX, and 156 °C in IVM corresponded to the melting point of drug crystals. These peaks were also found the physical mixture. However, these peaks were not observed in NS formulations, indicating the change of the nature of drugs from crystalline forms to amorphous. This may be caused by the method of preparation of NS. It was previously reported that the bottom-up method was able to produce amorphous nanoparticles [41].

### 3.4. In Vitro Drug Release Study

**Figure 4:**
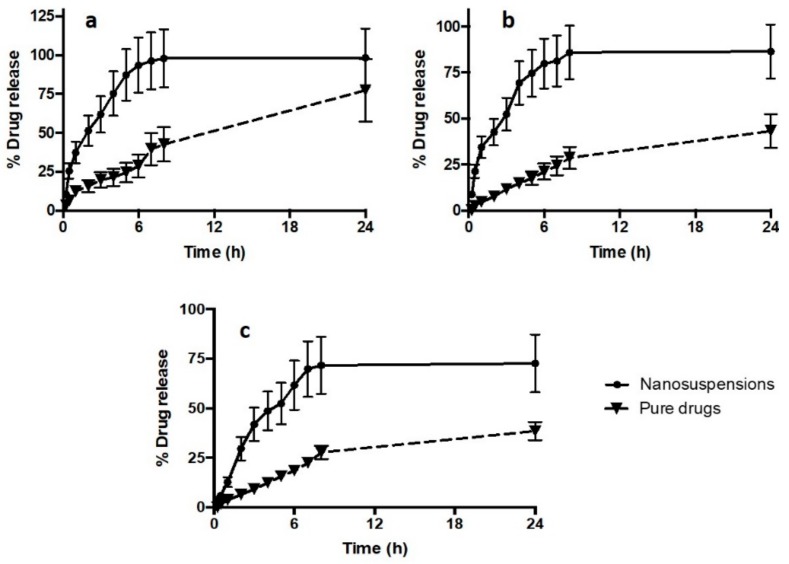
In vitro release profiles of (**a**) DOX NS, (**b**) ABZ-OX NS, and (**c**) IVM NS in comparison with the pure drug (means ± SD, *n* = 3).

The profiles of in vitro release of drug NS compared to pure drugs are illustrated in Figure 4. The release profiles of the drug NS revealed that the NS formulations were able to improve the release of all drugs. Specifically, after 24 h, the NS achieved 98.43 ± 18.70%, 86.45 ± 14.70%, and 72 ± 14.60% drug releases for DOX, ABZ-OX, and IVM NS, respectively. In contrast, only 77.43 ± 20.12%, 43.23 ± 9.08%, and 38.46 ± 8.17% of drugs were released in case of pure DOX, ABZ-OX, and IVM, respectively. Despite the lower drug release of pure DOX compared to DOX NS, no significant difference (*p*-value = 0.2562; not significant statistically) was found between these release profiles after 24 h. However, with respect to the release profile of ABZ-OX and IVM, drug release profiles from NS were found to be significantly higher (*p*-value = 0.0123; *p*-value < 0.05 and *p*-value = 0.0181; *p*-value < 0.05 for ABZ-OX and IVM formulations, respectively) than that of the pure drugs. The release profiles clearly showed the rapid diffusion rate of drugs from nanoparticles as a result of the increase in surface areas after reduction of the particle sizes to nano-size dispersions. Therefore, it is important to bear in mind that lymphatic uptake of the nanoparticle drugs should be achieved before the dissolution of the NS in the skin layers. However, the rapid dissolution of NS is unlikely to occur in the skin as the volume of interstitial is much lower than the volume of release media utilized in this study.

In order to investigate the kinetic modelling and release mechanism of NS, the release profiles were fitted to several kinetic models. The kinetic modelling profiles of all drugs are shown in Table 1. The appropriate release model was selected based on the value of the correlation coefficient of each model. The release profiles of all drugs exhibited best fit to first order model. This model describes that the drug release from the formulation matrix depends on the drug concentration within the matrix [30,45].

### 3.5. MN Formulations

**Figure 5:**
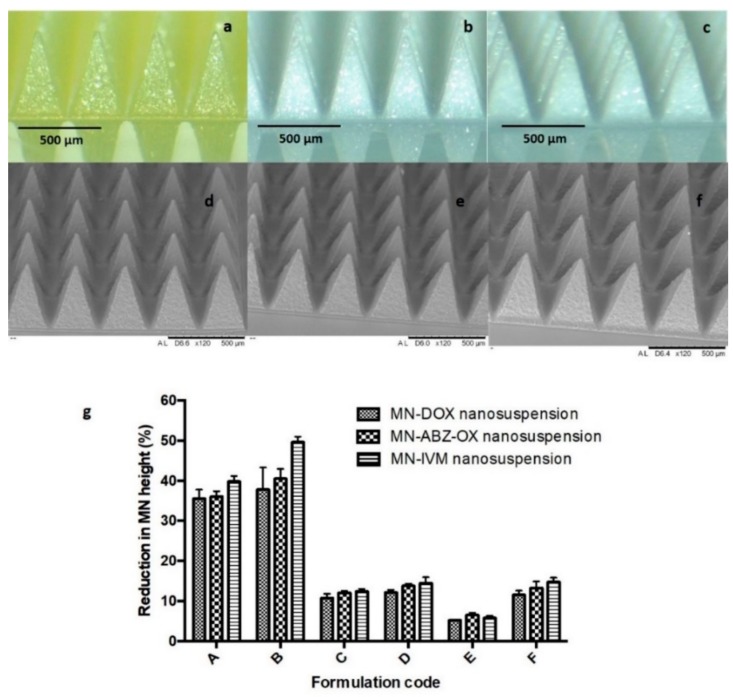
Light microscope images of the MN of Formulation E containing (**a**) 30% *w*/*w* of lyophilized NS of DOX, 15% *w*/*w* of PVA, and 5% *w*/*w* of PVP; (**b**) 30% *w*/*w* of lyophilized NS of ABZ-OX, 15% *w*/*w* of PVA, and 10% *w*/*w* of PVP and (**c**) 30% *w*/*w* of lyophilized NS of IVM, 15% *w*/*w* of PVA, and 10% *w*/*w* of PVP (C); SEM images of the MN containing drug NS of (**d**) DOX, (**e**) ABZ-OX, and (**f**) IVM (F); (**g**) Comparison of the percentage of height reduction of MN needles formulations containing 20% *w*/*w* of lyophilized NS of drugs and 50% *w*/*w* of PVP (formulation code A in each formulation); 30% of lyophilized NS of drugs and 50% of PVP (formulation code B in each formulation); 20% *w*/*w* of lyophilized NS of drugs and 15% *w*/*w* of PVA (formulation code C in each formulation); 30% *w*/*w* of lyophilized NS of drugs and 15% *w*/*w* of PVA (formulation code D in each formulation); 30% *w*/*w* of lyophilized NS of drugs and 15% *w*/*w* of PVA and 5% *w*/*w* of PVP (formulation code E in DOX formulation); 40% *w*/*w* of lyophilized NS of drugs and 15% *w*/*w* of PVA and 10% *w*/*w* of PVP (formulation code F in each formulation) (means ± SD, *n* = 3).

Several biocompatible polymers were used to fabricate the dissolving MN formulation in an attempt to load high concentrations of lyophilized NS. Many water-soluble, biocompatible polymers, such as PVA [46], PVP [47], HA [32], and Gantrez^®^ copolymers [48] have been widely used as polymers in the preparation of dissolving MNs. As described in formulation composition previously in Section 2.6, a plethora of different MN formulations were prepared to achieve the highest possible drug loading and analyzed with the results of the present study showing that all MNs prepared exhibited homogenous polymer blends with the resultant MN having sharp needle tips. All formulations were supported by dry baseplate prepared from 15% *w*/*w* PVP (MW 360 kDa) and 1.5% *w*/*w* glycerol. The morphology of selected MNs examined by light microscope and SEM are shown in Figure 5a–f. In addition to this, MNs containing free drug were also prepared, in order to allow their comparison with MN containing the drug NS. However, as the hydrophobic drugs did not dissolve in the aqueous environments of the MN polymers, the incorporation of these free drugs into MNs did not result in homogeneous distribution of the drug in the arrays and as such, MN lacked any inherent mechanical strength and so could not be used in further experiments.

### 3.6. Mechanical and Insertion Properties of MNs

The capability of a MN array to be effectively inserted is critical to its use, as the *stratum corneum* must be penetrated for the MN array to have its effect. Incorporation of drug substances including NS into the polymeric solution in the fabrication of a dissolving MN array can create either a weakening or strengthening effect on the MNs [49]. Therefore, mechanical tests must be carried out as an integral part of initial formulation studies for dissolving MN arrays.

Mechanical and insertion studies were carried out on all of the MN generated in this study. The percentage height reductions of needles on the various MN arrays were determined after application of forces of 32 N/array, equitable to manual compression force [33]. Figure 5g shows the percentage of height reduction of needles on MN formulated from each of the three candidate formulations. The results obtained here revealed that the formulation containing the combination of PVP and PVA with the drug loading of 30% *w*/*w* (Formulation E) exhibited the smallest percentage of height reduction of needles, which was < 10%. The percentage reduction of the needle heights for Formulation E of DOX, containing 30% *w*/*w* of lyophilized NS, 15% *w*/*w* of PVA, and 5% *w*/*w* of PVP was significantly lower (*p*-value < 0.05) compared to all other formulations. In ABZ-OX and IVM formulations, the percentage reduction of the needle heights for Formulation E containing 30% *w*/*w* of lyophilized NS, 15% *w*/*w* of PVA, and 10% *w*/*w* of PVP was significantly lower (*p*-value < 0.05) compared to all other formulations. In contrast, Formulation A and B containing only PVP and drug NS showed the highest percentage reduction of the needle heights with the percentage of height reduction of >30%, indicating poor mechanical properties. Moreover, Formulation C and D containing only PVA and drug NS exhibited the percentage of height reduction of >10%. The increase of drug loading to 40% *w*/*w* (Formulation F) resulted in a lack of mechanical strength, despite the homogenous and sharp needles formed. Therefore, Formulation E of each drug was selected for further studies. In addition to this, it was found that the baseplate used to support the MNs needles did not show any change after the compression, indicating that the baseplate had sufficient mechanical strength. The same force was applied to candidate MN formulations to evaluate the insertion ability of the MNs, using Parafilm^®^M as a skin model, as previously described [33].

**Figure 6:**
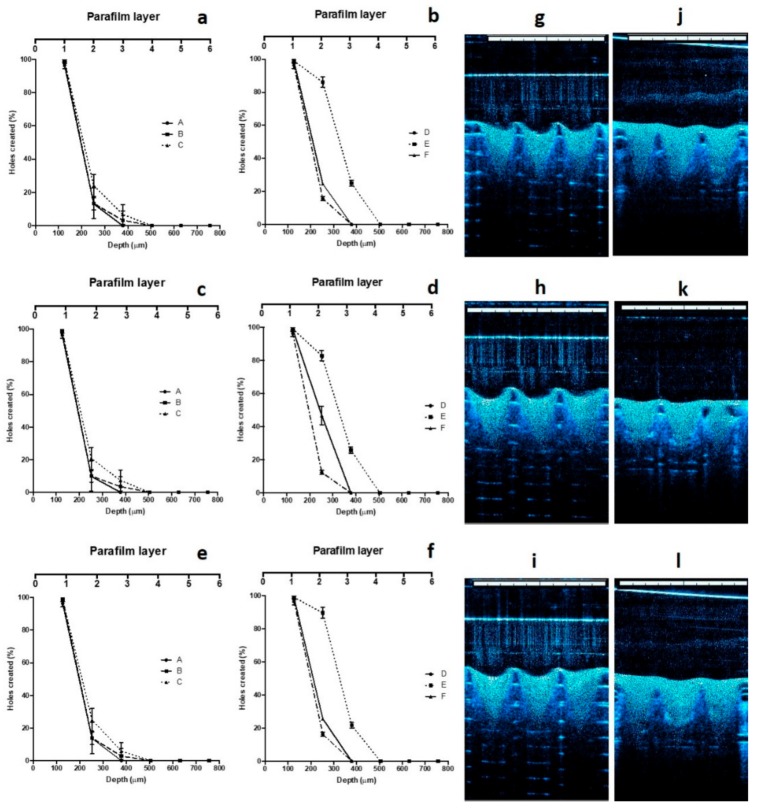
Number of Parafilm^®^M layers inserted, approximate insertion depths and percentage of holes created in Parafilm^®^M layer using an insertion force of 32 N/array using the Texture Analyzer for MN formulations containing DOX NS (**a**,**b**), ABZ-OX NS (**c**,**d**), and IVM NS (**e**,**f**) (means ± SD, *n* = 3). Representative optical coherence tomography (OCT) images of MNs containing (**g**) DOX NS, (**h**) ABZ-OX NS, and (**i**) IVM NS following insertion into Parafilm^®^M film. Representative OCT images of MNs containing (**j**) DOX NS, (**k**) ABZ-OX NS, and (**l**) IVM NS following insertion into full-thickness porcine skin. The white scale bar represents a length of 1 mm.

The results of the skin insertion study were in close agreement with those of the mechanical strength studies. In this evaluation, the same three candidate formulations were able to penetrate three layers of Parafilm^®^M. Figure 6a–f shows the insertion ability of the MNs presented as the number of holes created in each layer of Parafilm^®^M. The mean thickness of each layer is approximately 126 μm. Therefore, the MNs inserted up to 378 μm, relating to approximately 75.6% of the MN height. These results suggest that the needle lengths which inserted into the skin model was similar to a study reported previously [32]. Moreover, these values were similar with previous studies illustrating the insertion of polymeric MNs into human skin [50]. In addition to this, OCT was also employed in insertion studies. The selected formulations were evaluated in terms of insertion ability in both Parafilm^®^M and full-thickness porcine skin. Several studies have shown the advantages of this technique in the investigation of MN insertion in Parafilm^®^M and skin [26,32]. The results are exhibited in Figure 6g–l. MN penetration depth of Formulation E in Parafilm^®^M were found to be 369.12 ± 18.43 μm, 371.88 ± 19.19 μm, and 370.17 ± 17.13 μm for DOX, ABZ-OX, and IVM formulations, respectively. These insertion depths were in close agreement with those observed in similar studies carried out in full-thickness, showing depths of 368.17 ± 16.33 μm, 372.03 ± 19.87 μm, and 369.22 ± 13.93 μm for DOX, ABZ-OX, and IVM formulations, respectively.

### 3.7. Drug Content Located in the Needles

Upon drying, the amount of drug located in each array was found to be 1.00 ± 0.15 mg, 0.87 ± 0.09 mg, and 0.86 ± 0.07 mg for DOX, ABZ-OX and IVM, respectively.

### 3.8. In Vitro Dermatokinetic, Skin Distribution and Deposition Studies

**Figure 7:**
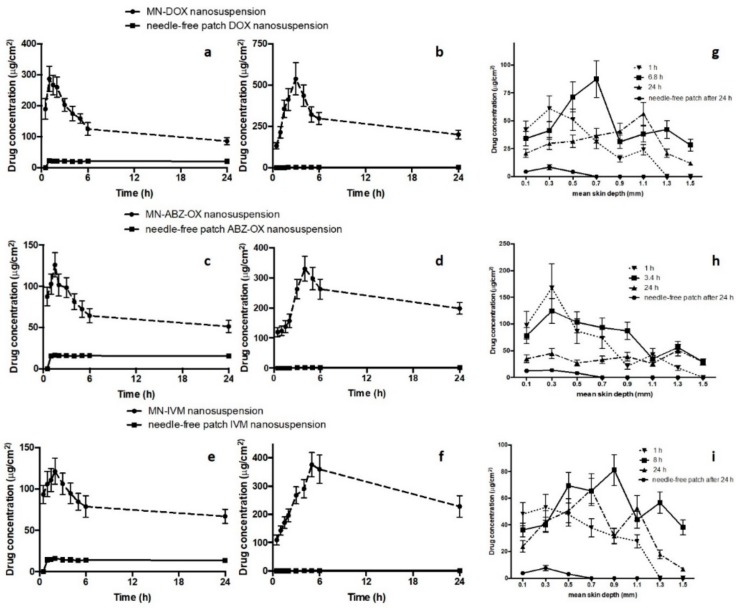
The drug concentration and time profile of DOX in epidermis (**a**) and dermis (**b**), DEC in epidermis (**c**) and dermis (**d**), and ABZ-OX in epidermis (**e**) and dermis (**f**) layers of excised full-thickness neonatal porcine skin, following the application of MN, in comparison with the application of free-needle patches (means ± S.D., *n* = 3). The concentrations of drugs in a different layer of neonatal porcine skin, following the application of MN containing (**g**) DOX NS; (**h**) ABZ-OX NS, and (**i**) IVM NS, in comparison with the free-needle patch (means ± S.D., *n* = 3).

Dermatokinetic studies were then carried out in order to evaluate the kinetic profile of drugs following MNs application in epidermis and dermis layer. Several studies have been conducted to investigate dermatokinetic profiles of drug delivery systems [34,51]. However, to the best of our knowledge, the work presented here is the first study investigating the dermatokinetic profile of drugs following MNs administration. The results show that the concentration of drugs in epidermis and dermis layer was significantly improved by using dissolving MNs compared to the needle-free patch (*p*-value < 0.05). Upon insertion of the MN arrays into the skin, the needles absorb the interstitial fluid and hence this fluid hydrates the polymer. Therefore, the polymer hydration and dissolution help the moving of the drug NS, which is then able to penetrate into the skin. Figure 7a–f describe the comparison of epidermal and dermal drug profile following MNs application in comparison with the needle-free patch.

The results of the DOX NS dermatokinetic profile after MNs administration indicated that the mean peak skin concentration (*C*_max_) in the epidermis was 266.31 ± 42.73 µg/cm^3^ within 1.28 ± 0.23 h. In the dermis layer, the maximum concentration of 409.63 ± 54.11 µg/cm^3^ was found after 3.47 ± 0.49 h. The AUC_0–24_ of DOX in the epidermis and dermis were 1978.90 ± 232.31 h.µg/cm^3^ and 6806.79 ± 790.54 h.µg/cm^3^, respectively. After MNs administration of ABZ-OX NS, 101.78 ± 21.32 µg/cm^3^ was found as the maximum concentration within 1.19 ± 0.31 h in the epidermis. The *C*_max_ in dermis was 300.34 ± 54.79 µg/cm^3^ after 6.88 ± 0.88 h. Furthermore, the AUC_0-24_ of 1547.91 ± 203.43 h.µg/cm^3^ and 5869.17 ± 598.34 h.µg/cm^3^ were found in epidermis and dermis, respectively. With respect to IVM NS profiles in the epidermis, the *t*_max_ was found to be 1.37 ± 0.29 h with a concentration of 108.02 ± 19.71 µg/cm^3^. In the dermis layer, *C*_max_ was found to be 372.07 ± 49.98 µg/cm^3^ after 8.01 ± 1.23 h. The AUC_0-24_ in epidermis and dermis were 2018.92 ± 342.32 h.µg/cm^3^ and 7122.40 ± 784.09 h.µg/cm^3^, respectively. In addition, the time required for the drugs to be released from the MNs (lag time) in the dermis were 0.294 ± 0.02 h, 0.354 ± 0.02 h, and 0.512 ± 0.04 h for DOX, ABZ-OX, and IVM, respectively. The values of *C*_max_, AUC_0__–__24_, permeation rate and elimination rate in the epidermis was found to be significantly higher (*p*-value < 0.05 each) than in dermis. In all cases, apart from *t*_max_, the values of all dermatokinetic parameters of drugs after MNs application were significantly higher (*p*-value < 0.05) than needle-free patch application in epidermis and dermis. Considering the drug amount in the actual needles of the arrays, 29.29 ± 4.65%, 31.54 ± 5.35%, and 34.54 ± 4.98% of DOX, ABZ-OX, and IVM were retained in the dermis after 24 h administration.

The results presented here revealed that the incorporation of drugs in NS into dissolving MN arrays significantly enhanced the delivery of those drug particles into the skin and led to the retention of the drug particles in the dermis layer, a site replete with lymphatic capillaries [52] and hence, could potentially target the *Wolbachia* and macrofilariae in the lymph nodes [3]. In addition, the drugs that were not retained in the dermis layer might be taken up by the blood capillaries, which could potentially kill the *Wolbachia* and microfilariae in the bloodstream [7]. A variety of published studies have demonstrated that nanoparticles of ~100 nm accumulate in lymph nodes [53,54,55]. Furthermore, work carried out in our research group demonstrated that incorporation of rhodhamine B-encapsulated nanoparticles of <100 nm into dissolving MNs led to the delivery of this dye to the lymph nodes of mice [56]. Accordingly, we propose that this combination delivery system could be used to target the relevant drugs to the anatomic sites of filarial nematodes.

The skin distribution and deposition of all drugs were then investigated in order to measure the distribution of the drug in full-thickness porcine skin. Figure 7g–i depict the amount of DOX, ABZ-OX, and IVM detected per cm^3^ in a range different depths of skin after three application times, namely, 1 h, *t*_max_ of dermis dermatokinetic profiles and 24 h. A close agreement results with the dermatokinetic study were obtained in this study, showing that the distribution of all drugs was significantly improved using MNs compared to needle-free patch. In the case of DOX distribution, as can be observed in Figure 7g, after 1 h, the DOX concentration rose significantly between 0.1 and 0.3 mm depths. This concentration declined significantly at a mean depth of 0.9 mm. The drug was not detected at a depth of 1.3 mm. After 6.8 h, the drug concentration reached a peak at a depth of 0.7 mm and was detected up to a depth of 1.5 mm. Finally, after 24 h, it was found that the drug was still detected in a depth of 1.5 mm and hit the highest point in a depth of 1.1 mm. With respect to ABZ-OX distribution (Figure 7h), there was a sharp increase in drug concentration between 0.1 mm and 0.3 mm depth after 1 h and 3.4 h. Furthermore, the drug concentration in the skin decreased dramatically and reaching the lowest concentration at a depth of 1.5 mm and 1.3 mm after 1 h and 3.4 h, respectively. The drug was not detected at 1.5 mm following a 1 h application time. After 24 h, the drug concentration remained unchanged between a depth of 0.1 mm and 1.1 mm. The maximum drug concentration was found in 1.3 mm depth and the drug concentration slightly decreased in a depth of 1.5 mm. In terms of IVM distribution (Figure 7i), 1 h application time exhibited the same trend as the ABZ-OX concentration after 1 h. The maximum concentration was found in a depth of 0.9 mm and detected until 1.5 mm after 8 h. After 24 h, the concentration increased from a depth of 0.1 mm to 0.7 mm and despite slight fluctuation, the drug concentration declined significantly until a depth of 1.5 mm. On the other hand, all drugs were only detected until 0.5 mm after 24 h application of the needle-free patch, indicating the ability of MNs to improve the penetration of the drugs. In all cases, it was found that the drugs were detected in the deeper layers of the skin than MNs height (500 µm), representing the movement of drugs in the skin. The great distribution of drugs after intradermal administration using MNs in combination with NS approach could potentially lead to the high possibility of lymphatic capillaries in dermis layer to take up the particles. With respect to the concentration of these drugs required to kill filarial nematodes, it was reported that 40 µg/mL of DOX [12], 26.53 µg/mL of ABZ [57], and 170 µg/mL of IVM [58] were able to kill *Brugia malayi* in vitro after 7 days, 16 days, and 4 days, respectively. These concentrations were around 2–10 times lower than the *C*_max_ obtained in the dermatokinetic study. However, further studies are now required to determine the concentrations of drugs which reach the infection site in vivo.

Taken together, the results presented here, combined with evidence reported in other published studies, indicate that the unique delivery approach of combining NS into dissolving MN, leads to successful intradermal delivery and drug retention in the dermis. The overriding benefit of the facilitated delivery approach we have reported here, compared to conventional oral administration, lies in the capacity for drug retention in the skin which could potentially lead to targeting of the lymphatic system when infected with filarial nematodes. Following on from these findings, further studies are now necessitated to investigate the in vivo lymphatic delivery capabilities of this system, in order to investigate the rate of which the NS is taken by the lymphatic system.

## 4. Conclusions

This study investigated the potential of dissolving MNs, used in combination with NS, to assist in the targeted delivery of DOX, ABZ-OX, and IVM to the dermis layer of skin. Several optimization steps were carried out to develop the NS formulation. PVP and SLS, utilized as stabilizers for DOX, and PF127 used as a stabilizer for ABZ-OX and IVM resulted in the successful formulation of NS with small particle sizes (<100 nm) and negative charges. The utilization of these NS enhanced the release of the drugs in vitro with the results of the dermatokinetic studies providing proof of principle for the intradermal delivery of antifilariasis drugs which could, in time, have positive implications in the clinical treatment of LF. Before this novel delivery system can achieve patient benefit, however, further comprehensive studies are required, including in vivo pharmacokinetic, lymphatic uptake, and pharmacodynamic studies in an appropriate model system. Furthermore, patient usability and acceptability must be determined in order to fully exploit the potential applications of this approach.

## Figures and Tables

**Table 1:** Kinetic modelling of DOX, ABZ-OX, and IVM release from NS formulations.

Formulation	*R*^2^ Value of Kinetic Model					*n*
	Zero Order	First Order	Higuchi	Hixcon-Crowell	Korsmeyer-Peppas	
DOX NS	0.4360	0.9311	0.5900	0.7032	0.7983	0.311
ABZ-OX NS	0.4486	0.9461	0.6049	0.7611	0.8054	0.314
IVM NS	0.4963	0.9759	0.76	0.8076	0.7891	0.391

## Supplementary Materials

The following are available online at https://www.mdpi.com/1999-4923/11/7/346/s1, Table S1: The experimental design of DOX, ABZ-OX, and IVM NS, Table S2: Central composite experimental design and the response values for the DOX NS formulation, Table S3: Central composite experimental design and the response values for the ABZ-OX NS formulation, Table S4: Central composite experimental design and the response values for the IVM NS formulation, Table S5: Results of fit statistical analysis for the all responses of NS formulations, Table S6: Predicted and observed responses of the optimized NS formulations, Figure S1: Particle size, PDI and zeta potential of DOX NS prepared using PVP, PVA, NaCMC, HPMC, Pluronic^®^ F127, F128, F68, and SLS in the initial screening process (means ± SD, *n* = 3), Figure S2: Particle size, PDI and zeta potential of ABZ-OX NS prepared using PVP, PVA, NaCMC, HPMC, Pluronic^®^ F127, F128, F68, and SLS in the initial screening process (means ± SD, *n* = 3), Figure S3: Particle size, PDI and zeta potential of IVM NS prepared using PVP, PVA, NaCMC, HPMC, Pluronic^®^ F127, F128, F68, and SLS in the initial screening process (means ± SD, *n* = 3).
