# Supplementary material for: Enhanced Intradermal Delivery of Nanosuspensions of Antifilariasis Drugs Using Dissolving Microneedles: A Proof of Concept Study
Source: Pharmaceutics. 2019 Jul 17;11(7):346. doi: 10.3390/pharmaceutics11070346 (PMC6680801; doi:10.3390/pharmaceutics11070346)
Supplement: Supplementary file 1 [file pharmaceutics-11-00346-s001.pdf]

pharmaceutics

MDPI

Supplementary Materials: Enhanced Intradermal Delivery of Nanosuspensions of Antifilariasis Drugs Using Dissolving Microneedles: A Proof of Concept Study

Andi Dian Permana, Maelíosa T.C. McCrudden and Ryan F. Donnelly

Table S1. The experimental design of DOX, ABZ-OX and IVM NS.

Factors	Level				
	-alpha	low	medium	high	+alpha
Drug concentration (mg)	14.64	25	50	75	85.35
Stabilizer concentration (% *w*/*v*)					
DOX	0.29	0.5	1	1.5	1.71
ABZ-OX and IVM	0.39	0.5	0.75	1	1.1
Antisolvent volume (mL)	0.94	2.5	6.25	10	11.53
Sonication time (min)	2.93	5	10	15	17.07

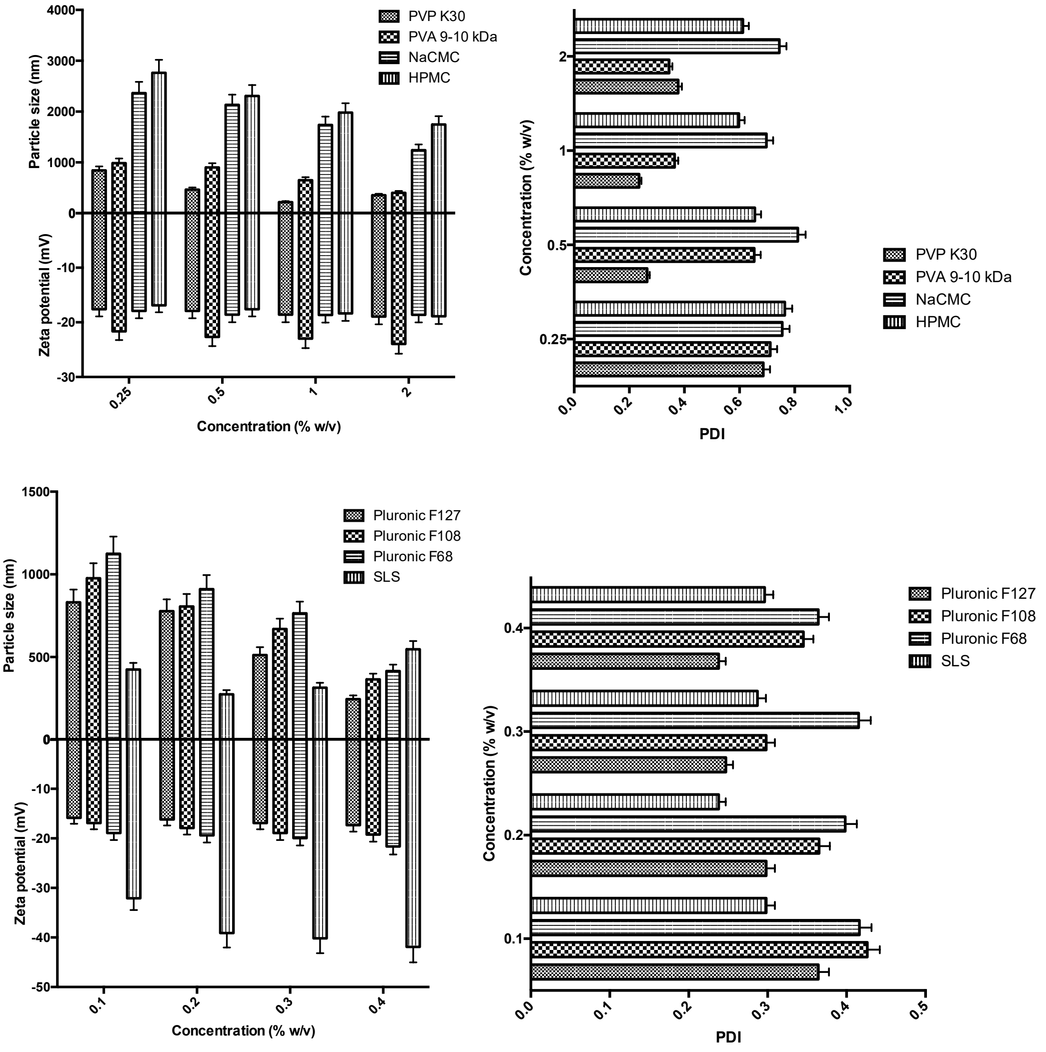

Figure S1. Particle size, PDI and zeta potential of DOX NS prepared using PVP, PVA, NaCMC, HPMC, Pluronic ® F127, F128, F68 and SLS in the initial screening process (means ± SD, *n* = 3).

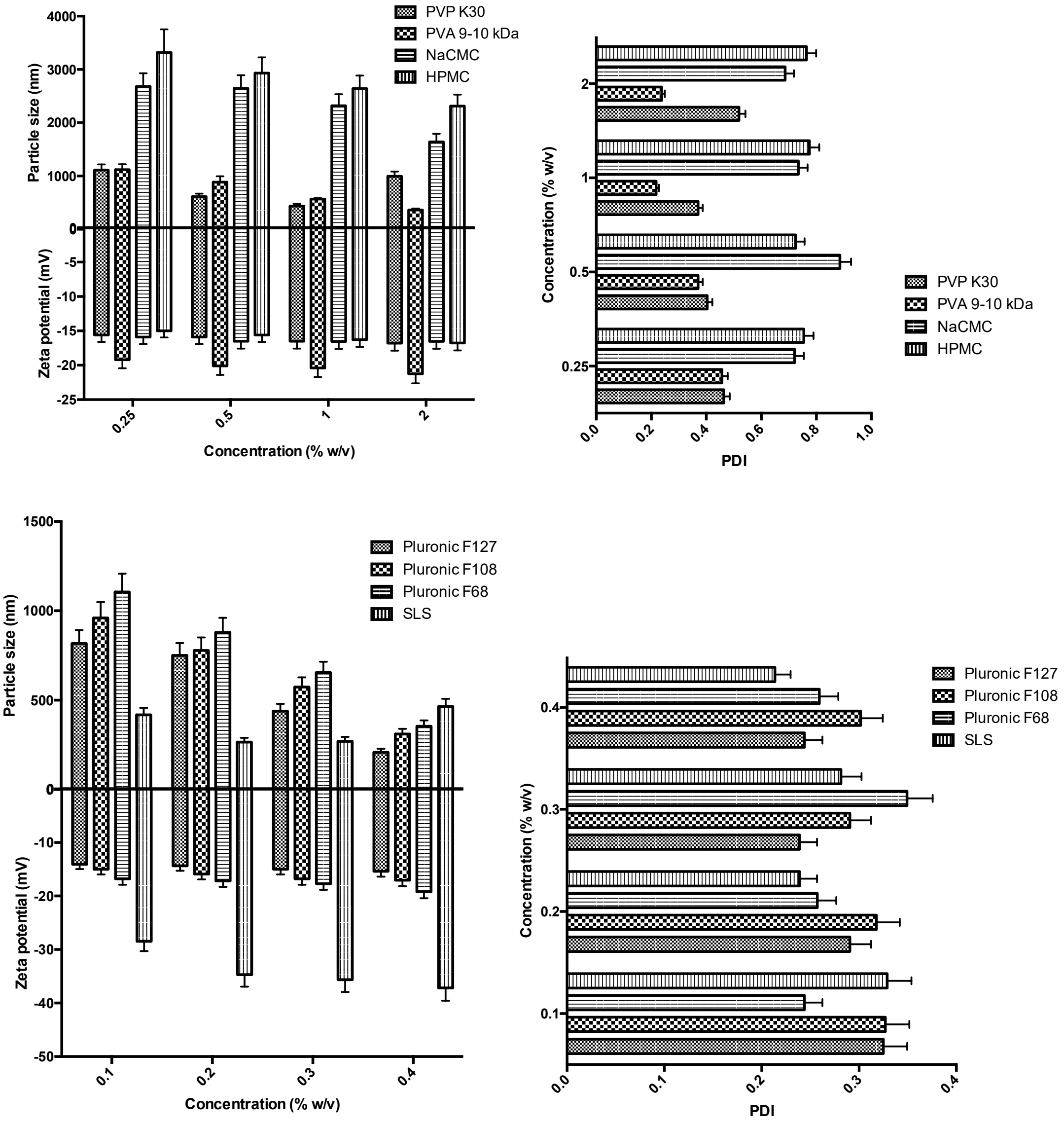

Figure S2. Particle size, PDI and zeta potential of ABZ-OX NS prepared using PVP, PVA, NaCMC, HPMC, Pluronic ® F127, F128, F68 and SLS in the initial screening process (means ± SD, n = 3).

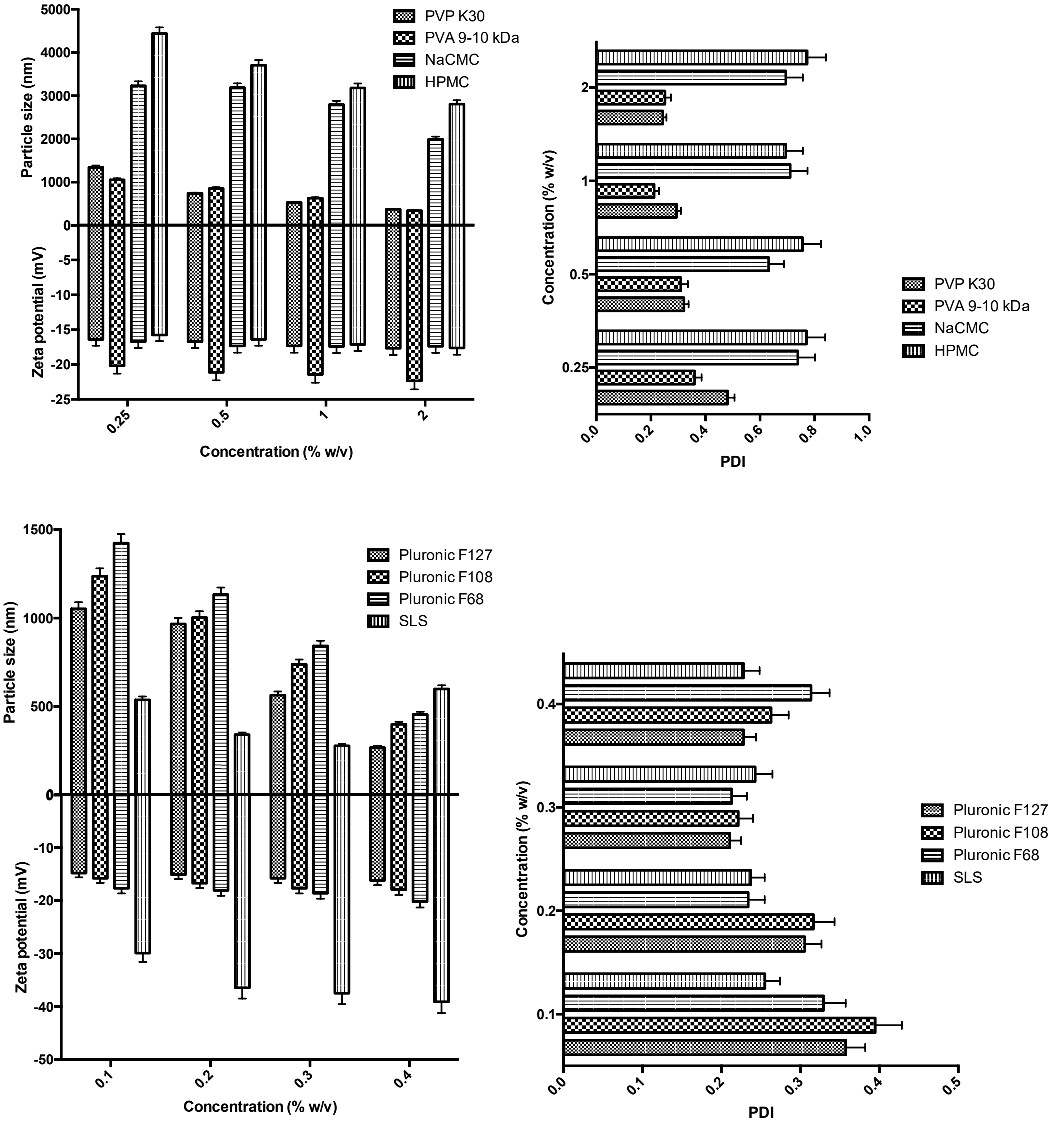

Figure S3. Particle size, PDI and zeta potential of IVM NS prepared using PVP, PVA, NaCMC, HPMC, Pluronic ® F127, F128, F68 and SLS in the initial screening process (means ± SD, *n* = 3).

Table S2. Central composite experimental design and the response values for the DOX NS formulation.

Run	Factors				Responses		
	DOX concentration (mg)	PVP concentration (% w/v)	Antisolvent volume (mL)	Sonication time (min)	Particle size (nm)	PDI	Zeta potential (mV)
1	75	1.5	2.5	15	333.43	0.443	−24.12
2	50	1	0.95	10	698.54	0.699	−26.88
3	50	0.29	6.25	10	321.65	0.432	−35.43
4	85.35	1	6.25	10	530.43	0.598	−26.66
5	75	1.5	10	15	238.77	0.365	−24.08
6	25	1.5	2.5	15	123.49	0.287	−23.43
7	50	1	6.25	2.93	135.43	0.299	−27.12
8	75	1.5	10	5	279.76	0.398	−24.21
9	25	1.5	10	15	85.43	0.203	−23.87
10	75	1.5	2.5	5	601.92	0.623	−23.98
11	50	1.71	6.25	10	105.65	0.262	−23.99
12	25	0.5	10	15	132.32	0.291	−31.27
13	25	0.5	2.5	5	423.98	0.512	−31.29
14	25	0.5	10	5	154.33	0.312	−30.98
15	50	1	11.55	10	98.54	0.243	−26.87
16	75	0.5	2.5	15	960.43	0.798	−29.98
17	75	0.5	10	15	363.87	0.459	−30.13
18	25	0.5	2.5	15	320.22	0.412	−31.32
19	25	1.5	2.5	5	214.2	0.337	−23.12
20	75	0.5	2.5	5	1136.27	0.834	−29.73
21	75	0.5	10	5	438.29	0.532	−29.78
22	14.64	1	6.25	10	87.43	0.206	−27.43
23	25	1.5	10	5	104.32	0.259	−23.43
24	50	1	6.25	17.07	132.87	0.298	−26.91

Table S3. Central composite experimental design and the response values for the ABZ-OX NS formulation.

Run	Factors				Responses		
	ABZ-OX concentration (mg)	Pluronic concentration (% *w/v*)	Antisolvent volume (mL)	Sonication time (min)	Particle size (nm)	PDI	Zeta potential (mV)
1	25	0.5	10	15	130.34	0.283	−16.72
2	75	0.5	2.5	5	987.41	0.789	−15.98
3	75	0.5	10	15	429.09	0.503	−16.20
4	75	1	2.5	5	589.28	0.589	−12.89
5	14.64	0.75	6.25	10	86.12	0.196	−14.67
6	50	0.75	6.25	2.93	133.40	0.284	−14.58
7	50	1.10	6.25	10	103.49	0.249	−12.90
8	75	1	2.5	15	326.43	0.425	−12.97
9	50	0.75	6.25	17.07	130.08	0.276	−14.47
10	25	1	2.5	5	210.99	0.324	−12.36
11	50	0.75	11.55	10	96.47	0.231	−14.45
12	25	1	10	15	84.15	0.193	−12.76
13	25	0.5	2.5	5	417.62	0.484	−16.73
14	75	0.5	10	5	356.23	0.434	−16.01
15	85.36	0.75	6.25	10	519.29	0.566	−14.33
16	75	1	10	15	233.76	0.350	−12.95
17	75	0.5	2.5	15	940.26	0.755	−16.12
18	25	1	10	5	102.76	0.246	−12.53
19	25	0.5	2.5	15	315.42	0.396	−16.75
20	25	1	2.5	15	121.64	0.273	−12.53
21	75	1	10	5	273.89	0.382	−13.02
22	25	0.5	10	5	152.02	0.296	−16.57
23	50	0.75	0.95	10	683.87	0.661	−14.45
24	50	0.40	6.25	10	498.83	0.415	−19.05

Table S4. Central composite experimental design and the response values for the IVM NS formulation.

Run	Factors				Responses		
	IVM concentration (mg)	Pluronic concentration (% *w/v*)	Antisolvent volume (mL)	Sonication time (min)	Particle size (nm)	PDI	Zeta potential (mV)
1	25	0.5	10	15	136.85	0.297	−17.56
2	75	0.5	2.5	5	1168.03	0.828	−16.78
3	75	0.5	10	15	450.54	0.528	−17.01
4	75	1	2.5	5	618.74	0.619	−13.54
5	14.64	0.75	6.25	10	90.42	0.205	−15.40
6	50	0.75	6.25	2.93	140.07	0.298	−15.31
7	50	1.10	6.25	10	108.60	0.261	−13.54
8	75	1	2.5	15	342.75	0.447	−13.62
9	50	0.75	6.25	17.07	136.58	0.290	−15.19
10	25	1	2.5	5	220.10	0.340	−12.98
11	50	0.75	11.55	10	100.64	0.242	−15.17
12	25	1	10	15	87.78	0.202	−13.40
13	25	0.5	2.5	5	435.66	0.509	−17.57
14	75	0.5	10	5	371.62	0.456	−16.81
15	85.36	0.75	6.25	10	541.72	0.594	−15.24
16	75	1	10	15	243.85	0.368	−13.76
17	75	0.5	2.5	15	980.88	0.793	−17.13
18	25	1	10	5	107.19	0.257	−13.32
19	25	0.5	2.5	15	331.57	0.416	−17.80
20	25	1	2.5	15	127.87	0.287	−13.32
21	75	1	10	5	287.91	0.402	−13.84
22	25	0.5	10	5	159.80	0.312	−17.61
23	50	0.75	0.95	10	718.89	0.695	−15.36
24	50	0.40	6.25	10	333.05	0.436	−20.25

Table S5. Results of fit statistical analysis for the all responses of NS formulations.

	Responses	R^2	Adjusted R^2	Predicted R^2	Std. Dev.	Adeq Precision
DOX	Particle size	0.9700	0.9233	0.7865	77.61	17.0647
	PDI	0.9585	0.8938	0.7173	0.0588	13.9614
	Zeta potential	0.9936	0.9837	0.9577	0.4345	33.5281
ABZ-OX	Particle size	0.9686	0.9198	0.7775	77.62	16.4328
	PDI	0.9607	0.8996	0.7310	0.0539	14.0324
	Zeta potential	0.9934	0.9831	0.9561	0.2376	33.2903
IVM	Particle size	0.9682	0.9187	0.7742	81.93	16.3066
	PDI	0.9606	0.8993	0.7302	0.0567	14.0159
	Zeta potential	0.9907	0.9762	0.9387	0.2997	28.396

Table S6. Predicted and observed responses of the optimized NS formulations.

	Factors				Responses	Predicted	Observed	Bias (%)
	Drug concentration (mg)	Stabilizer concentration (% *w/v*)	Antisolvent volume (mL)	Sonication time (min)				
DOX	50.69	1.271	9.243 (containing 0.2% *w/v* SLS)	5.431	Particle size (nm)	96.26	98.87 ± 9.77	−2.71
					PDI	0.276	0.258 ± 0.01	6.52
					Zeta potential (mV)	−25.58	−28.94 ± 1.98	-13.13
ABZ-OX	50.753	0.981	6.66	6.623	Particle size (nm)	92.32	96.53 ± 8.43	−4.56
					PDI	0.261	0.251 ± 0.02	3.83
					Zeta potential (mV)	−13.6	−14.32 ± 1.32	−5.29
IVM	50.106	0.993	6.746	5.655	Particle size (nm)	94.27	98.12 ± 7.76	−4.08
					PDI	0.272	0.233 ± 0.02	14.33
					Zeta potential (mV)	−14.18	−15.07 ± 0.95	−6.27
